# Mixed-Dimensional Naphthylmethylammoinium-Methylammonium Lead Iodide Perovskites with Improved Thermal Stability

**DOI:** 10.1038/s41598-019-57015-4

**Published:** 2020-01-16

**Authors:** Bhumika Chaudhary, Teck M. Koh, Benny Febriansyah, Annalisa Bruno, Nripan Mathews, Subodh G. Mhaisalkar, Cesare Soci

**Affiliations:** 1 Interdisciplinary Graduate School, Energy Research Institute @ Nanyang Technological University (ERI@N), Research Techno Plaza, X-Frontiers Block, Level 5, 50 Nanyang Drive, 637553 Singapore, Singapore; 2 Energy Research Institute @ Nanyang Technological University (ERI@N), Research Techno Plaza, X-Frontiers Block, Level 5, 50 Nanyang Drive, 637553 Singapore, Singapore; 3 School of Materials Science and Engineering, Nanyang Technological University, 50 Nanyang Avenue, 639798 Singapore, Singapore; 4 Division of Physics and Applied Physics School of Physical and Mathematical Sciences, Nanyang Technological University, 21 Nanyang Link, 637371 Singapore, Singapore

**Keywords:** Chemistry, Energy science and technology, Engineering, Materials science, Nanoscience and technology

## Abstract

Metal halide perovskite solar cells, despite achieving high power conversion efficiency (PCE), need to demonstrate high stability prior to be considered for industrialization. Prolonged exposure to heat, light, and moisture is known to deteriorate the perovskite material owing to the breakdown of the crystal structure into its non-photoactive components. In this study, we show that by combining the organic ligand 1-naphthylmethylammoinium iodide (NMAI) with methylammonium (MA) to form a mixed dimensional (NMA)_2_(MA)_*n*−1_Pb_*n*_I_3*n*+1_ perovskite the optical, crystallographic and morphological properties of the newly formed mixed dimensional perovskite films under thermal ageing can be retained. Indeed, under thermal ageing at 85 °C, the best performing (NMA)_2_(MA)_*n*−1_Pb_*n*_I_3*n*+1_ perovskites films show a stable morphology, a low PbI_2_ formation rate and a significantly reduced variation of both MA-specific vibrational modes and fluorescence lifetimes as compared to the pristine MAPbI_3_ films. These results highlight the role of the bulky NMA^+^ organic cation in mixed dimensional perovskites to both inhibit the MA^+^ diffusion and reduce the material defects, which act as non-radiative recombination centres. As a result, the thermal stability of metal halide perovskites has been substantially improved.

## Introduction

Within the past few years, intensive research efforts in perovskite composition and structure modification^1,2^, morphology improvement, interfacial engineering, additive incorporation, and optimization of device architecture^3–8^ have substantially improved the perovskite materials and perovskite solar cells (PSCs) performances^9^. The three dimensional (3D) MAPbI_3_ perovskite, thanks to its high charge carrier mobility, high absorption, and long charge carrier diffusion length together with the low-cost and facile processability^3,10–12^, has been deeply explored in PSCs reaching a power conversion efficiency (PCE) of ~21%^13–16^. However MAPbI_3_ suffers from long-term stability due to the presence of hygroscopic CH_3_NH_3_^+^ cation^17^. Other environmental factors, such as exposure to oxygen, heat, and light, further aggravate material degradation^18,19^, thus collectively reducing reliability and impeding commercialization^20–22^.

To make the perovskite technology industrially appealing and suitable for outdoor applications, it is necessary to enhance the material longevity under external environmental stimuli. Addressing this, compositional engineering approaches have been explored to make stable perovskite materials; for instance, incorporation of formamidinium (FA) and cesium (Cs) into MAPbI_3_ perovskites was shown to improve stability up to several hours to weeks^4,6,23,24^. Concurrently, hydrophobic surface passivation of the active layer, device encapsulation by water-resistive polymers and incorporation of cross-linking hydrophobic additives were also employed to further enhance stability^25–27^. Although those strategies are promising to improve stability against moisture and oxygen, they are not effective to improve stability against heat. Thermal degradation of the MAPbI_3_ perovskite, where high-temperature annealing induces movement of ionic species and distortion of the crystal lattice, remains little understood^28–30^. Thus, since the crucial issue of intrinsic thermal instability of MAPbI_3_ cannot be addressed at the device level, it should be tackled by structural modification of the active material^30^.

Recently, mixed dimensional perovskites of the type (M)_2_(CH_3_NH_3_)_*n*−1_Pb_*n*_X_3*n*+1_ (M = long/bulky alkyl ammonium cation, X = halide anion) have drawn great attention for their enhanced moisture stability^31,32^. For instance, the combination of MA cations and bulky alkyl-ammonium cations (M), such as phenylethylammonium (PEA)^33^, poly(ethyleneimine) (PEI)^34^, ethylenediamine (EDA)^35^, butylamine (BA)^36^, or cyclopropylamine (CA)^37^, fluorous based cations (3-(Nonafluoro-tert-butyloxy)propylamine hydroiodide)^38^ and aminovaleric acid iodide (AVAI)^39^ to form hybrid 2D/3D or layered perovskites yielded to a good moisture stability^40^. Encouragingly, Cs doping in (BA)_2_(MA)*n*Pb_*n*_I_3*n*+1_ was also shown to improve the thermal stability of mixed dimensional perovskite devices, with relatively high PCE of 13.7%^41^. Thus, the incorporation of alkyl-ammonium cations provides an excellent opportunity to study and improve the inherent stability of hybrid perovskites.

Till now, most stability studies have focused on the behavior of device PCE under constant illumination, or as a function of operating temperature^39,42^. In this approach, several factors contribute to the overall PSCs stability, including degradation of the active layer, the hole transporting material (HTM), and all the interfaces in the multilayer device stack^43^. To pinpoint the processes that induce thermal instability of the active perovskite material, it is crucial to isolate device components and interlayer effects^30^. Recent reports have highlighted that organic ammonium cations embedded with conjugated moieties are useful to enhance the charge transport and stability (light and/or moisture) of PSCs^44–46^. Thus, in an attempt to enhance the perovskite thermal stability, we have combined the large and conjugated organic cation 1-naphthyl-methylammonium (NMA) and methylammonium (MA) cation to form mixed dimensional (NMA)_2_(MA)_*n*_Pb_*n*_I_3*n*+1_ perovskites. The NMA organic cation was chosen over other linear or less conjugated cations^40,47^, as its strong π-π conjugation facilitates the formation of 2D perovskite and allows an improved charge transport across the layered structure due to the strong interaction between the NMA fused aromatic functionalities. Moreover, we expect the NMA cation to have a conductivity comparable to similar naphthalene based bulky molecules^48^. The later one has indeed demonstrated to improve the out-of-plane conductivity in layered perovskite^48^. NMA has been recently combined with formamidinium (FA) and cesium (Cs) cations to form stable and efficient LED devices^47,49^, while its employment in PSCs and its effect on thermal stability has not been studied previously.

In this work, we found that, compared to standard MAPbI_3_, the higher order *n* NMA based mixed dimensional perovskites morphological, optical, and structural properties are inherently tolerant to the thermal ageing even at elevated temperatures (85° and/or 150°). This is also reflected by the good thermal stability of an entire solar cell stack, without compromising the overall device performance (PCE ~17%).

## Results and Discussion

**Figure 1:**
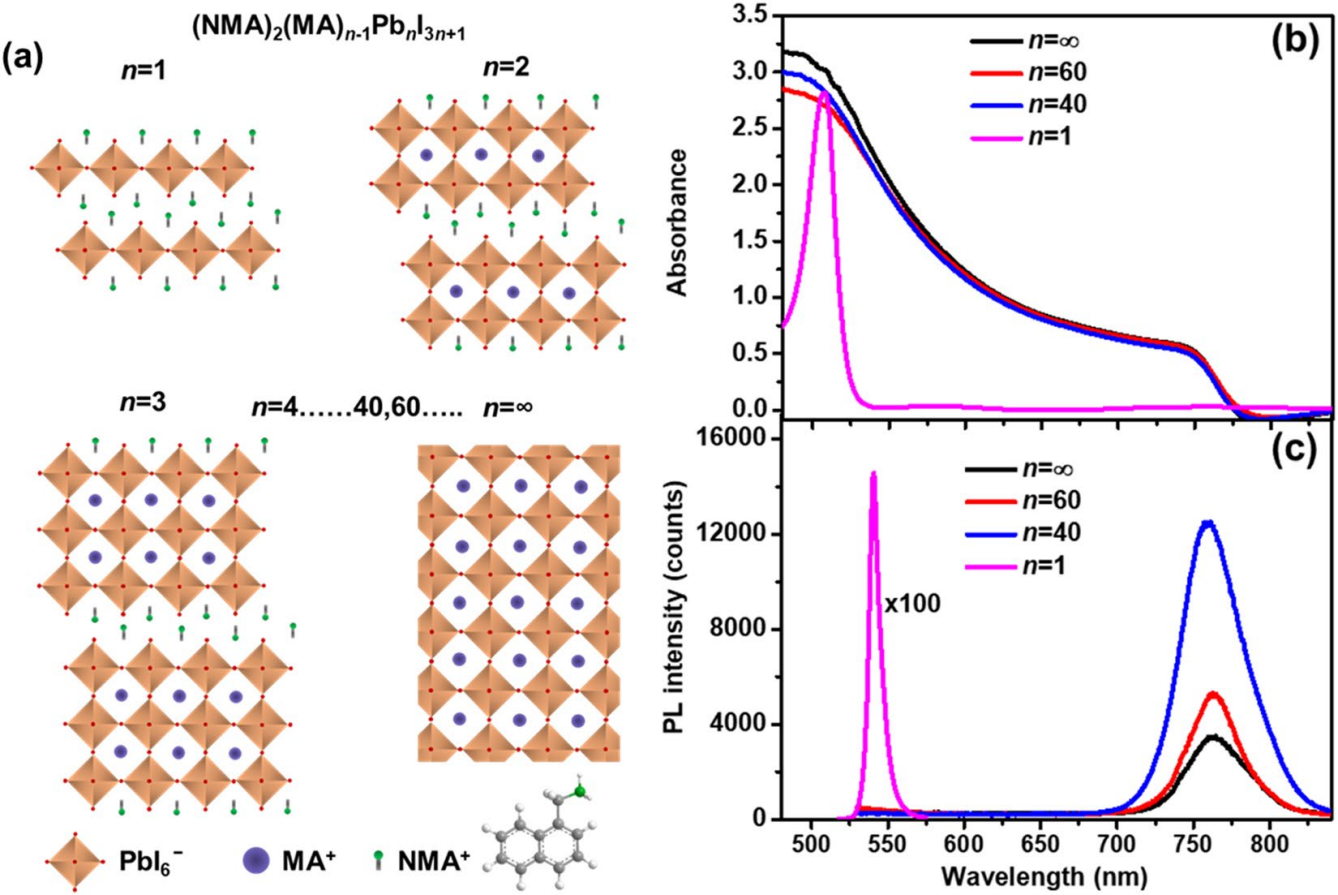
Structure and optical properties of mixed dimensional perovskites: (**a**) schematic illustration of (NMA)_2_(MA)_*n*−1_Pb_n_I_3*n*+1_ perovskite structures with different stoichiometry, where *n* = 1 corresponds to pure 2D and *n* = ∞ to 3D perovskites; (**b**) absorption spectra of *n* = ∞, dimensionally controlled (*n* = 60, 40), and 2D (NMA)_2_PbI_4_ (*n* = 1) perovskites; (**c**) photoluminescence spectra of the corresponding perovskite films (*n* = 1, 60, 40 and ∞) deposited on glass. The *n* values of mixed dimensional perovskite are based on the composition of precursor solution, calculated by using (NMA)_2_(MA)_*n*−1_Pb_*n*_I_3*n*+1_ stoichiometry.

Previous reports have highlighted how organic ammonium cations can significantly to enhance the stability of PSCs^44–46^. In this study, we investigated a series of mixed dimensional perovskites (NMA)_2_(MA)_*n*−1_Pb_*n*_I_3*n*+1_ and analyse systematically the impact of the NMA ligand on thermal stability of MAPbI_3_ perovskite. Pure two-dimensional (2D) (*n* = 1), low (*n* ≤ 10) and high (*n* = 20, 40, 60) order *n* mixed dimensional, and 3D perovskites (*n* = ∞) films were deposited on quartz or glass substrates. Detailed procedures and conditions to obtain perovskites with different dimensionalities are summarised in the Methods section. Here, the *n* values of mixed dimensional perovskite are defined based on the composition of the precursor solution, calculated by using (NMA)_2_(MA)_*n*−1_Pb_*n*_I_3*n* + 1_ stoichiometry. A schematic of the resulting mixed dimensional perovskite structures with stoichiometry (NMA)_2_(MA)_*n*−1_Pb_*n*_I_3*n*+1_ is shown in Fig. 1a (*n* = 1 corresponds to pure 2D and *n* = ∞ to 3D perovskites). The formation of mixed dimensional structures was confirmed by glancing incident XRD and NMR analysis. The glancing incident XRD patterns of higher order *n* = 60, 40, 20 perovskite thin films exhibit major sharp peak of two-theta reflection at 14.2°, 28.3° and 31.8°, corresponding to the (110), (220) and (310) orientations, respectively, alike the XRD peaks of the 3D perovskite structure (*n* = ∞, Fig. S1b). As different amounts of NMAI salt are added to form mixed dimensional perovskites, no shift in the peak of typical tetragonal phase (110) is observed, indicating negligible volume expansion of the lattice in higher order *n* perovskites^50^. Moreover, the emergence of a new Bragg peak at a lower angle of 6.1° (Fig. S1c,d) is attributed to the formation of a layered structure, leading to the elongation of the crystal unit cell^50,51^. *n* ≤ 10 perovskites show higher diffraction at small angles (Fig. S1a), indicating that low *n* values facilitate the formation of low dimensional quasi 2D layered structures. Moreover, the diffraction peak intensity of the higher order *n* perovskites corresponding to (110) increases with the addition of NMAI bulkier cation, indicating an overall improvement of the crystallinity of mixed dimensional perovskites (Fig. S1b). ^1^H NMR studies additionally confirmed the presence of NMA cations in the resulting perovskite films. ^1^H NMR spectra of the (NMA)_2_(MA)_*n*−1_Pb_*n*_I_3*n*+1_ *n* = 40 and 60 samples, scraped from films with different NMA ratios and dissolved in the dimethyl sulfoxide-d_6_ (DMSO-d_6_), show that the actual the NMA:MA ratio in the perovskite film is quite close to the ratio in the precursor material (Fig. S3**)**. Peaks at 2.37 and 7.48 ppm chemical shift corresponds to the methyl and ammonium groups of the MA cation, respectively. Peaks in the range of 7.5–8.2 ppm are ascribed to aromatic protons of NMA, while the methylene linker of the cation brings about a peak at ~4.55 ppm. Due to their distinct chemical shift, the ratio between integral peaks of methyl (-CH_3_) in MA and methylene (-CH_2_) in NMA is used to estimate the relative amount of each cation in the high*-n* mixed dimensional perovskites. Simple calculations considering the relative number of protons of each cation molecule reveal that the measured NMA-MA ratios are 1:20 and 1:29 for *n* = 40 and *n* = 60, respectively. This is in very good agreement with the initial stoichiometry of cations in the precursor solution (1:19.5 and 1:28.5 for *n* = 40 and 60, respectively).

The variations in optical properties of the low-dimensional perovskite series after incorporation of the 2D barrier were subsequently analysed using absorption and photoluminescence, spectroscopy. While high order *n* mixed dimensional perovskites show an optical band edge around 780 nm, close to that of 3D MAPbI_3_, low dimensional perovskites (*n* ≤ 6) are characterized by the appearance of distinct excitonic absorption peaks in the 500–600 nm spectral region, which indicate the formation of mixed dimensional phases governed by the amount of NMAI ligand added (Fig. 1b and Fig. S4**)** for comparison of all dimensionalities). These trends are consistent with previous observations in other types of mixed dimensional perovskites^33^. The absorption properties of mixed dimensional perovskites are mirrored by their photoluminescence spectra (Fig. 1c), where high order *n* and 3D perovskites are characterized by broad luminescence centered around 770 nm with relatively small Stokes shift, while the 2D perovskite shows narrower emission spectrum, with much higher intensity and with significant Stokes shift, as expected for excitonic emission in low-dimensional systems^49,52^. The increase of band-edge photoluminescence in high order *n* perovskites upon addition of small amounts of bulky NMAI ligand is attributed to the reduction of non-radiative defect centers in the perovskites films, similarly as reported previously with other organic cations^53,54^.

After confirming the formation of mixed dimensional perovskites and determining their optical properties, we have analysed the thermal stability of higher order *n* mixed dimensional perovskites, as these retain optimal absorption characteristics for photovoltaic applications. To this end, perovskite films were directly deposited on glass to eliminate all probable degradation mechanisms related to the presence of additional layers and interfaces in the device stack, and the films were subjected to thermal ageing at 85 °C in an inert atmosphere (Ar-filled glove box) to eliminate oxygen or moisture-induced degradation. The effects of thermal ageing on morphology, absorption, crystallographic structure, vibrational properties (chemical bonding) and charge carrier lifetime were then evaluated.

**Figure 2:**
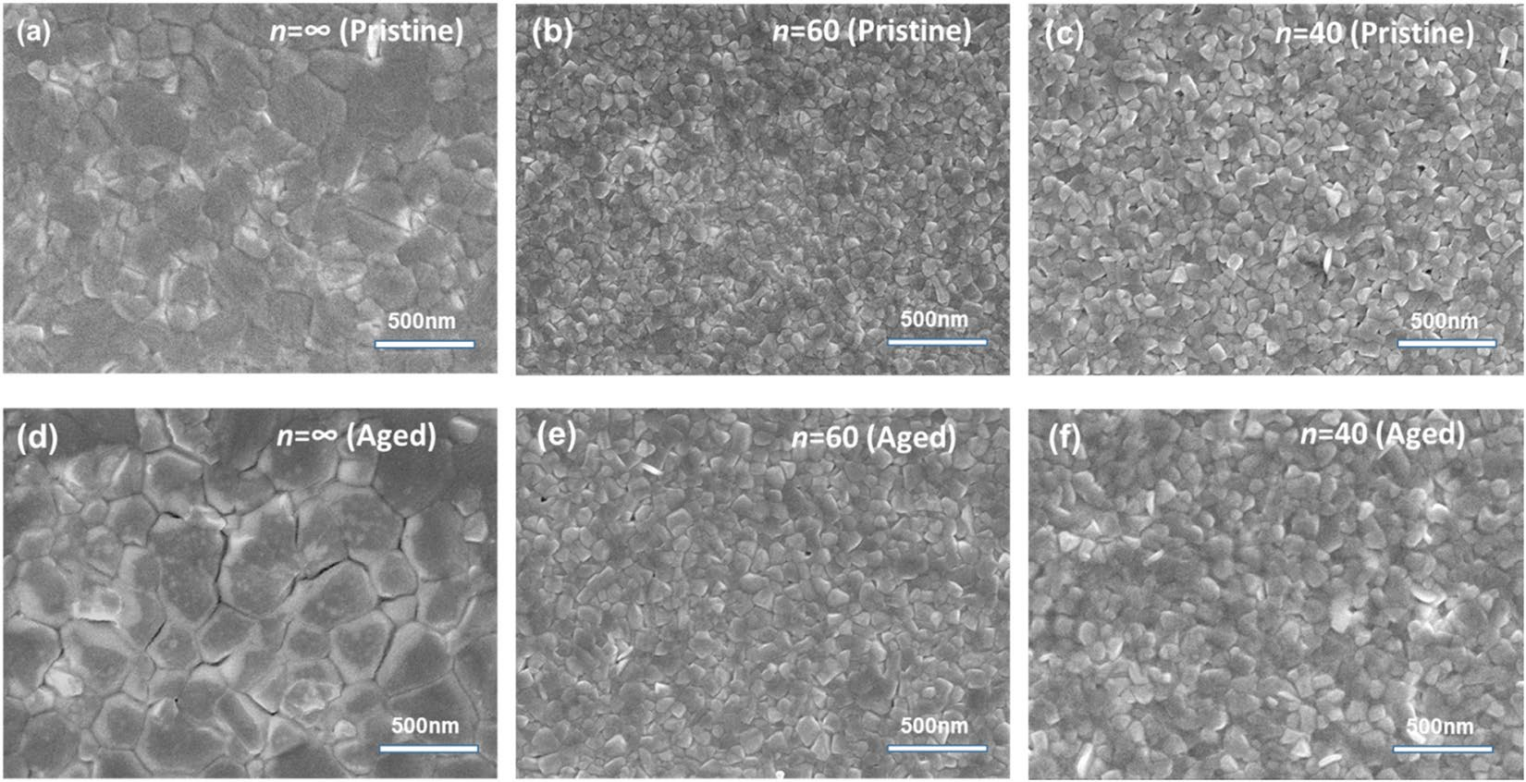
Scanning electron micrographs before and after thermal ageing at 85 °C for 4 hrs of pure MAPbI_3_ *n* = ∞ (**a,d**), mixed dimensional *n* = 60 (**b,e**) and *n* = 40 (**c,f**) perovskite thin films.

Uniform, pinhole-free, and large grains *n* = ∞ perovskite thin films, as shown in Fig. 2a, have been fabricated by using the anti-solvent dripping method^55^. After thermal ageing, the thin films showed enlarged grains (~50% larger, Fig. S5a) and several cracks at the grain boundaries, but none was observed in the bulk of perovskite grains (Fig. 2d). Previous reports have shown that decomposition of MAPbI_3_ into its non-photoactive components (CH_3_NH_3_I and PbI_2_) either by moisture, oxygen or heat, starts from the edges of crystal grains and not from the bulk^56^. Thus, we attribute the formation of cracks to the displacement of MA cations in the crystal upon thermally induced bond breakdown^24^.

The addition of NMA cation (*n* = 60 and 40) resulted in significantly different perovskite film morphology, leading to smaller and more compact grains, thus suggesting that incorporation of bulky organic cation significantly affects both crystal growth and morphology (Fig. 2b for *n* = 60 and Fig. 2c for *n* = 40). Moreover very differently to what has been observed for the *n* = ∞ perovskite, mixed dimensional perovskites thin films showed no cracks and a reduced increase of grain sizes (~30%) upon thermal ageing, indicating the resilience of higher order *n* mixed dimensional perovskite materials to thermal ageing (Fig. 2e and Fig. S5b for *n* = 60 and Fig. 2f and Fig. S5c for *n* = 40).

**Figure 3:**
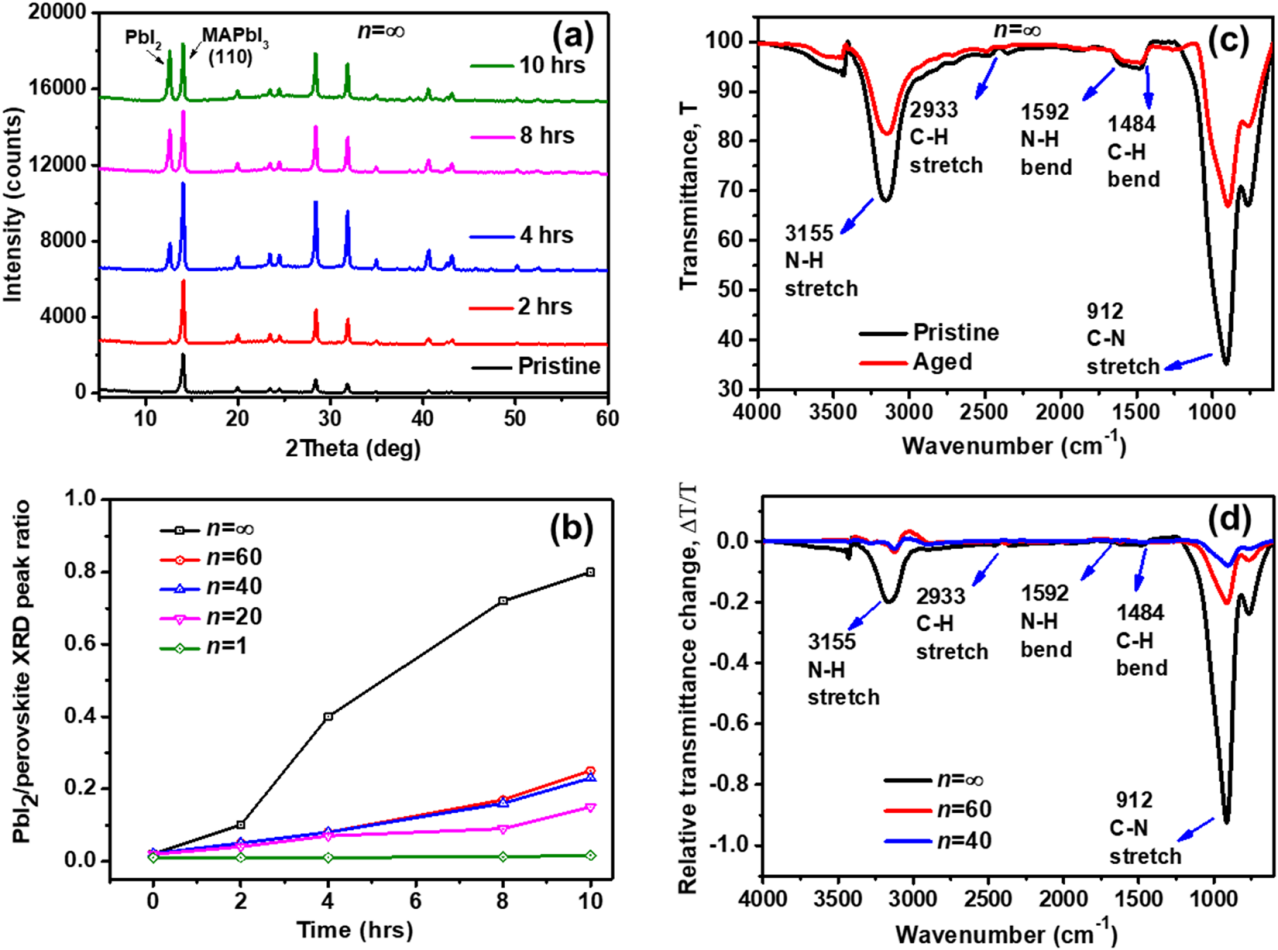
(**a**) Temporal evolution of the XRD spectra of *n* = ∞ perovskite films thermally aged at 85 °C in dark and in inert atmosphere for the period indicated (the pristine film was measured before ageing); (**b**) ratio between the PbI_2_ diffraction peak (2θ = 12.7°) and the main perovskite diffraction peak (2θ = 14.2°) intensities for 3D, mixed dimensional, and 2D perovskite films. (**c**) Pristine and aged *n* = ∞ Transmittance (T) spectra measured by a Fourier Transformed Infrared-Attenuated Total Reflectance (FTIR-ATR); **d**) relative transmittance change, ∆T/T of *n* = ∞, 60, 40 perovskite films after ageing for 4 hrs at 85 °C.

Detailed analysis of thermal ageing on perovskite crystal structure was conducted by glancing incident XRD diffractometry of the thin films. The X-ray diffractograms in Fig. 3a show clear structural changes of the pristine *n* = ∞ perovskite film after ageing at 85 °C for periods of 2, 4, 8 and 10 hours in an inert atmosphere. Thermal degradation of the *n* = ∞ structure evolves rapidly, as seen from the decrease of intensity of the perovskite main diffraction peak at 14.2° and by the appearance of the PbI_2_ peak at 12.6° just after 2 hrs thermal ageing. On the contrary, no substantial PbI_2_ peak could be observed in the diffraction patterns of the *n* = 60 and *n* = 40 perovskites even after 8 hours of heating (Fig. S6a,b**)**. Since the rate of PbI_2_ formation is directly correlated with the degradation of perovskite films, in Fig. 3b we compare the ratio between the PbI_2_ and the main perovskite diffraction peaks for structures with different dimensionality during the ageing process. The relative content of PbI_2_ in the *n* = ∞ perovskite increases more steeply over time than in the mixed dimensional perovskites **(**Fig. 3b), which provides direct evidence of the improved thermal stability of MA-NMA mixed dimensional perovskites. Furthermore, *in-situ* XRD measurements have been carried out to observe the continuous structural changes of perovskite films. The *in-situ* isothermal ageing of the *n* = ∞ and *n* = 60 thin films has been performed at a constant temperature of 85 °C under vacuum; eliminating the perovskite film exposure to other external stimuli (air and moisture). The XRD patterns over 16 hours ageing time for both perovskite films (*n* = ∞, 60) are shown in Fig. S7a,b. Moreover, Fig. S7c,d report a zoom-in between 12° to 15°, highlighting the slower formation of the PbI_2_ peak in the mixed dimensional perovskite consistently with improved stability. The PbI_2_/ perovskite ratio peak shows a much slower increase rate with the ageing time for the mixed dimensional *n* = 60 composition as compared to *n* = ∞ (MAPbI_3_) perovskite film, which clearly indicates the improved thermal stability of mixed dimensional perovskite film under constant thermal ageing process, Fig. S8.

Very similarly, XRD patterns of *n* = ∞ and *n* =  60 perovskites, at room temperature (RT) before and after the 16 hours isothermal ageing at 85 °C, Figure S9a,b, show a reduced PbI_2_ peak for the *n* =  60 perovskites, consistently indicating the improved thermal stability of mixed dimensional perovskite at elevated temperature. It is worth to note that both of *n* = ∞ and *n* =  60 perovskites show the phase transition from tetragonal to the cubic phase when the temperature reached 85 °C and the leftover perovskites return to their tetragonal phase after the long thermal ageing at 85 °C.

Attenuated Total Reflection-Fourier Transform Infrared (ATR-FTIR) spectroscopy was employed to monitor the changes in the vibrational modes of the organic cation associated with materials degradation upon thermal ageing (Fig. 3c,d**)**. The vibrational bands are characteristic of perovskite films containing the CH_3_NH_3_^+^ (MA) cation, for which the methyl (CH_3_−) functional group has C-H bending mode signature near 1480 cm^−1^ and C-H stretching mode at 2930 cm^−1^, along with the ammonium N-H and C-N stretching modes and N-H bending modes at around 3100, 900 and 1680 cm^−1^, respectively^20^. Upon thermal ageing, there is a clear reduction of the N-H and C-N stretching modes in the *n* = ∞ perovskite (Fig. 3c). This indicates a major change in the bonding of the lead halide framework with MA cation upon thermal ageing, which leads to the breakdown of CH_3_NH_3_PbI_3_. The relative change in the C-N stretching mode oscillator strength is significantly smaller in mixed dimensional perovskites *n* = 40, 60 (Fig. S10c,d) than in the *n* = ∞ perovskite (Fig. 3d), suggesting that the presence of bulky organic cations (NMAI) in the mixed dimensional perovskites could prevent the displacement of MA cations by passivating defects and grain boundaries which are primary pathways for ionic movement^57^. The large change of infrared vibrational modes indicates that the displacement of MA cations is more likely to occur in the *n* = ∞ perovskite compared to the high order *n* mixed dimensional perovskites. Prior reports have also suggested that ion movement is impaired in lower dimensional perovskite films by the physical action of the bulky organic spacers^58^. Thus, the stoichiometric ratio of the NMAI ligand to form higher order *n* mixed dimensional perovskites can reduce ionic motion by both, space-filling effects at grain boundaries and reductions of defects sites^58,59^, significantly improving the overall thermal stability of mixed dimensional perovskites.

**Figure 4:**
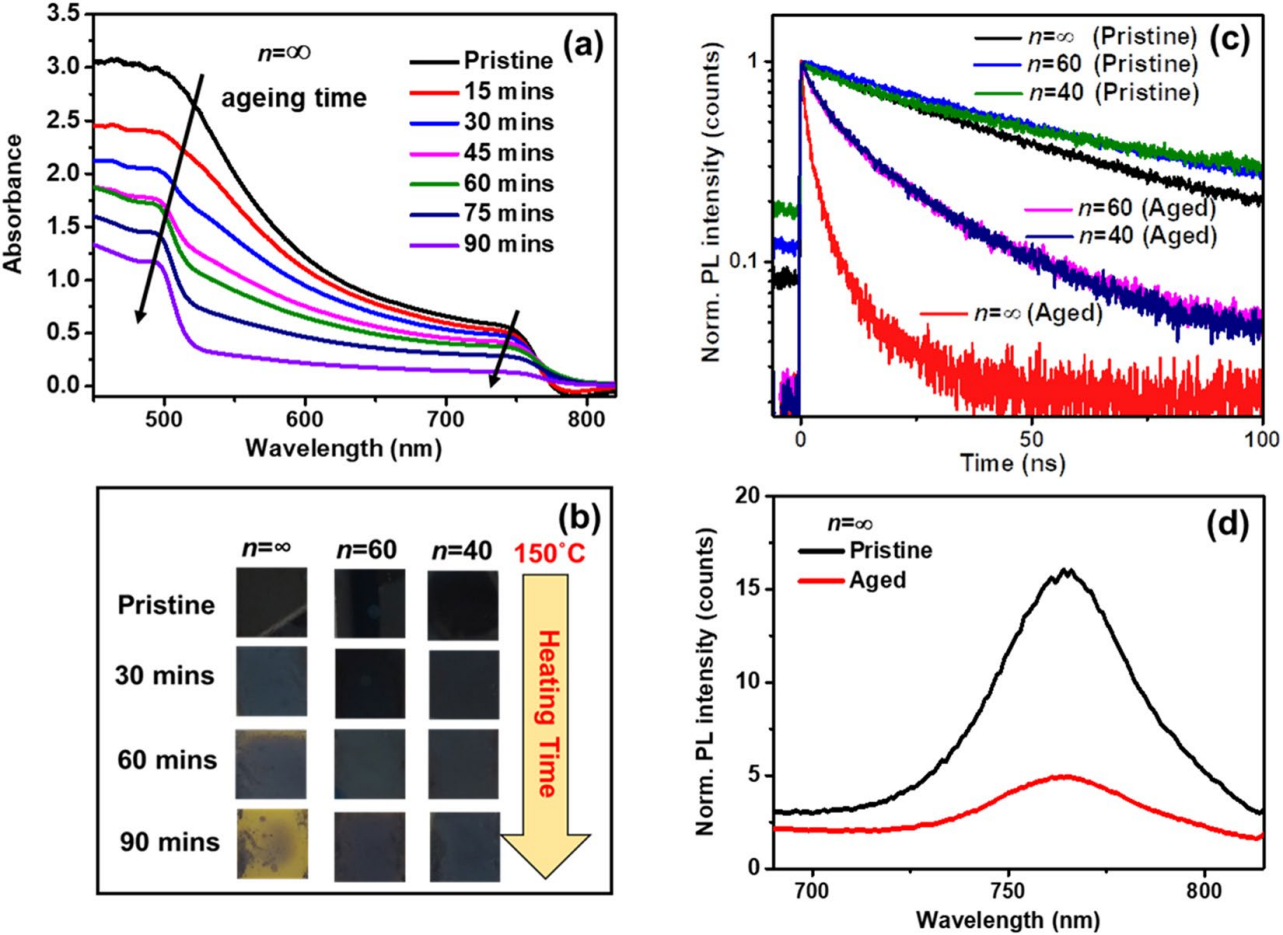
Effect of thermal ageing on the optical properties of high order mixed dimensional and 3D perovskites. (**a**) Time evolution of the absorption spectrum of the *n* = ∞ (3D MAPbI_3_) perovskite film upon heating at T = 150 °C and (**b**) the corresponding film colour variation. (**c**) Time-resolved photoluminescence decay dynamics of perovskite films with different dimensionality, aged at 85 °C for 4 hrs; (**d**) steady-state photoluminescence spectra of *n* = ∞ perovskite before and after ageing at 85 °C for 4 hrs.

Signs of thermal ageing are also manifested in the visible optical properties (absorption and photoluminescence) of the films. To check the thermal stability of perovskites beyond 85 °C, we heated the samples at 150 °C up to 90 minutes and monitored the variation of the absorption properties. The time evolution of the UV-vis absorption spectra of pristine MAPbI_3_ perovskite (*n* = ∞) upon thermal ageing is shown in Fig. 4a. Over time, the main absorption peak of the 3D perovskite at 780 nm significantly reduces, while the characteristic peak of PbI_2_ emerges from the background at 510 nm. This directly marks the progressive degradation of MAPbI_3_ at elevated temperature. On the other hand, with the addition of small amounts of NMA cation (*n* = 60 and 40), no significant PbI_2_ peak was detected in the optical absorption (Fig. S10a,b) under similar conditions. Moreover, degradation of the pristine perovskite film is manifestly shown by the film colour change from black to yellow (characteristic of PbI_2_) after 90 minutes. In contrast, the mixed dimensional perovskites containing the bulky cations hardly show any colour variation (Fig. 4b). These findings confirm the effectiveness of adding a small amount of NMAI, forming a mixed dimensional perovskite, to enhance the thermal stability of the films even at elevated temperatures, without affecting the bandgap of the perovskite material.

The time-resolved photoluminescence (TRPL) decay curves of pristine and aged perovskite films are shown in Fig. 4c. The fluorescence lifetimes of *n* = 60 and *n* = 40 perovskites are longer than in the 3D perovskite (fitting parameters for the measured fluorescence decay curves are summarized in Table S1). This is consistent with the reduction of non-radiative defect states inferred from the higher luminescence intensity of mixed dimensional perovskites already observed in Fig. 1c^53^. The increase of fluorescence lifetime saturates in mixed dimensional perovskites with *n* ≤ 40, likely due to the interplay between non-radiative defect reduction and the formation of crystallographic defects upon addition of stoichiometric amounts of NMAI to form mixed dimensional perovskite. Thermal ageing results in the reduction of both photoluminescence intensity (Fig. 4d) and fluorescence decay time (Fig. 4c) in the *n* = ∞ perovskite.

Conversely, the mixed dimensional perovskites display less significant variations under similar ageing conditions (Fig. 4c). The increase of non-radiative decay pathways upon ageing can be attributed to the formation of non-photoactive components upon degradation of the films, as indicated by optical and XRD analysis. As a matter of fact, such effects are greatly reduced in the mixed dimensional perovskites.

**Figure 5:**
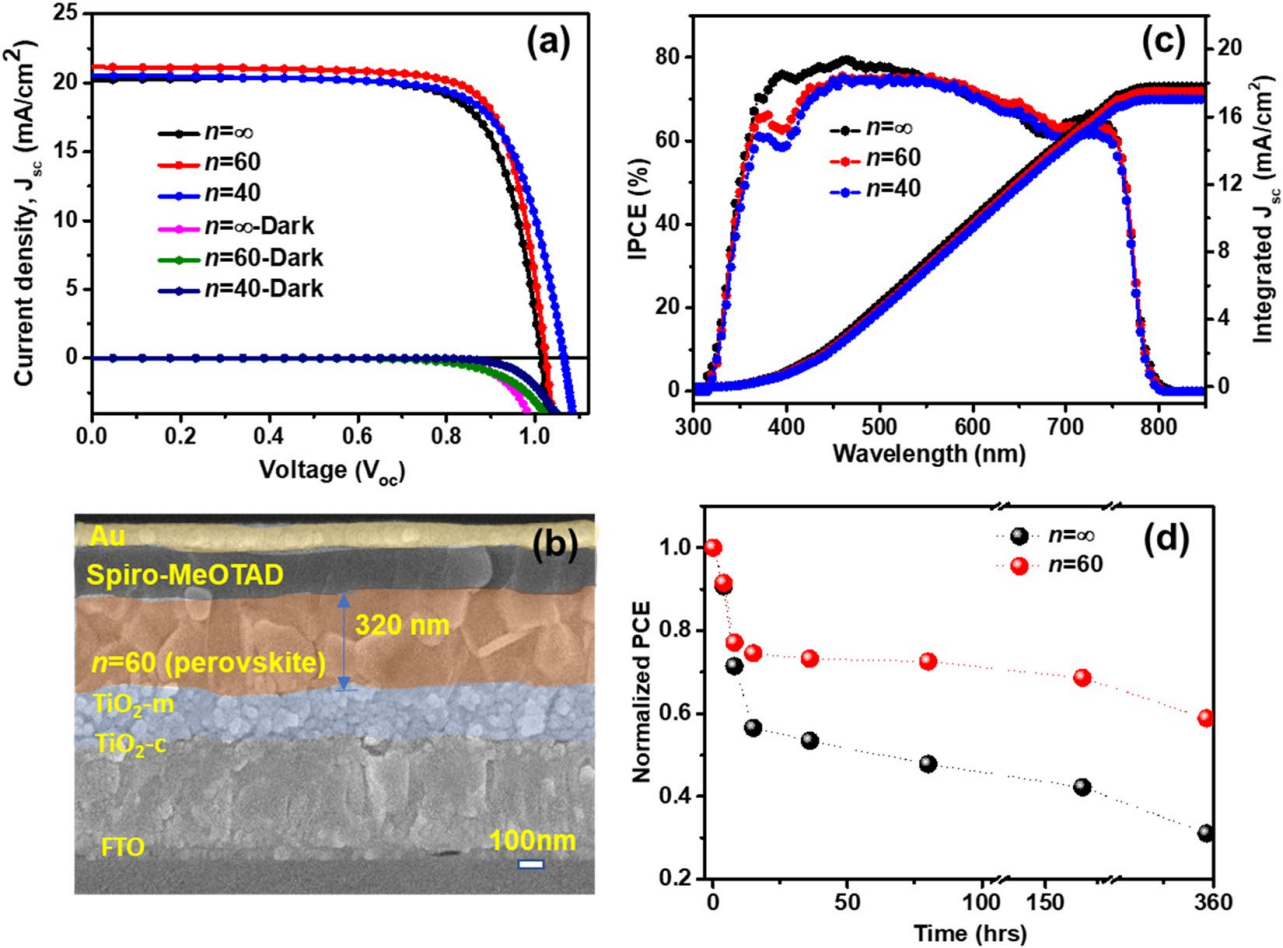
High order mixed dimensional and 3D PSCs. (**a**) Short circuit current density (J_sc_)−open circuit voltage (V_oc_) characteristics of the champion devices for *n* = ∞, 60, 40, (**b**) cross-section SEM image of dimensionally controlled *n* = 60 PSC, (**c**) corresponding IPCE spectra and (**d**) thermal stability test of encapsulated devices at 85 °C for 360 hrs.

We further fabricated PSCs using perovskite films of different dimensionality on a mesoporous device architecture (Fig. 5b) and compared their photovoltaic characteristics (see the Methods section for the detailed device fabrication procedure). Except for a slight increase in the thickness of the perovskite active layer, the overall mixed-dimensional perovskite device structure remains unaffected by the incorporation of the organic cation (NMAI) (Figs. 5b and S11). Solar cell characteristics were found to be well reproducible over 20 different devices (Fig. S13). The J-V characteristics of best-performing devices are shown in Fig. 5a (reverse scan), and their corresponding parameters are summarized in Table 1. The mixed dimensional perovskite devices (*n* = 40 and 60) show comparable performance to the 3D perovskite device. The *n* = 60 mixed dimensional perovskite device shows the highest efficiency (PCE~17%) amongst those fabricated, thanks to improvements in fill factor (FF = 78%) and open-circuit voltage (V_oc_ = 1.03). Since the optical bandgap does not vary significantly with incorporation of NMAI (Fig. S4), the systematic increase of V_oc_ in mixed dimensional perovskites (from 1.01 V in *n* = ∞ to 1.04 and 1.07 V in *n* = 60 and *n* = 40, respectively) is likely the result of the consistent reduction of defects by the bulky organic cations, which was found to reduce the non-radiative recombination centres. At the same time, the short circuit current density (J_sc_) of mixed-dimensional perovskite devices is overall preserved, while their IPCE reduces slightly in the short wavelength region corresponding to the absorption of TiO_2_, pointing to higher interfacial recombination at the mesoporous TiO_2_-mixed perovskite interface (Fig. 5c). The forward-reverse scan characteristics of the three types of devices are displayed in (Fig. S12a–c. Hysteresis is reduced in the higher order n mixed dimensional perovskite devices, indicating further effects of bulky organic ligand incorporation on ionic transport (Fig. S12).

**Table 1:** J-V characteristics of the best-performing device of *n* = ∞ and mixed dimensional perovskites (*n* = 60, 40).

(NMA)_2_(MA)_*n*−1_Pb_*n*_I_3*n*+1_	V_oc_ (V)	Jsc (mA cm^−2^)	Fill Factor (FF)	Efficiency (%)
*n =* ∞	1.01	20.6	76.6	15.9
*n =* 60	1.03	20.9	78.2	16.9
*n =* 40	1.07	20.5	73.3	16.1

The thermal stability of the encapsulated *n* = 60 mixed dimensional perovskite device was tested under a constant temperature of 85 °C and compared with the *n* = ∞ perovskite device (Fig. 5d). After an initial drop of efficiency, which does not correlate with the thermal behaviour of the individual perovskite layers and may therefore be due to degradation of the hole transporting layers in the device structure^60^, the *n* = 60 mixed dimensional perovskite device showed a significant improvement in overall stability, retaining 70% of its original PCE after 360 hrs. Conversely, the efficiency of the *n* = ∞ perovskite device dropped to 25% of its initial value under similar testing conditions. This shows that the enhanced thermal stability of mixed dimensional perovskite films, induced by incorporation of the bulky organic ligands, directly impacts thermal stability of solar cell devices, at an elevated temperature of 85 °C.

## Conclusions

In summary, we showed that the thermal stability of MAPbI_3_ perovskite is significantly improved by combining the 1-NMA organic ligand with MA to form mixed dimensional (NMA)_2_(MA)_*n*−1_Pb_*n*_I_3*n*+1_ perovskites. By studying the effects of thermal ageing on optical, crystallographic and morphological properties, the improvement in thermal stability has been attributed to the reduction of the traps and defects, which act as non-radiative recombination centers, by the bulky organic cations. Furthermore, the change of MA-specific vibrational modes in mixed-dimensional perovskite thin films and the reduction of electrical hysteresis in the corresponding solar cells indicate a reduction of ionic mobility which may also arise from space-filling at defects sites or at grain boundaries. Consequently, PSCs employing mixed dimensional perovskite as active layers show significant improvement in thermal stability: the best performing *n* = 60 PSCs retained 70% of their original PCE when heated at 85 °C for up to 360 hours, compared to the 25% PCE retention of a 3D perovskite control device. These results show how dimensional engineering of metal-halide perovskite films can substantially improve the thermal stability of photovoltaic devices under field-operating conditions, providing an additional pathway toward environmental stability and reliability of required for the timely commercialization of PSCs.

## Methods

### 1-Naphthylmethylammonium Iodide synthesis

The 1-naphthylmethylamine precursor (Sigma-Aldrich, 97%) (mL, mol etc) was dissolved in 10 mL of ethanol in round bottom flask before it was placed in the ice bath. The HI aqueous solution (Sigma-Aldrich, 99.99% 57 wt% in H_2_O) was then added dropwise during continues stirring of the solution, after which, the mixture was allowed to stir for another 2 hrs. The solvents were after that removed using a rotary evaporator. The solids, thus, obtained were filtered, washed with copious diethyl ether and recrystallized in ethanol to obtain a white powder (yield; 80%). Finally, the ammonium salts were dried under vacuum at 50 °C overnight, until the colorless powder precipitated. The precipitate was filtered out, washed thoroughly with diethyl ether and dried under vacuum. Recrystallization of dried crude was carried out in ethanol to obtain a white powder (yield ~80%). ^1^H NMR (Fig. S2) (400 MHz, DMSO-d_6_): δ 8.23 (s, ^3^H, NH_3_), 8.14 (d, ^1^H, ArH), 8.01 (t, ^2^H, ArH), 7.56–7.68 (m, ^4^H, ArH), 4.55 (s, ^2^H, CH_2_). ^13^C NMR (100 MHz, DMSO-d_6_): δ 133.2, 129.8, 128.7, 126.8, 125.4, 123.5, 39.4.

### Film formation

The 2D (*n* = 1), lower (≤10), higher (*n* = 20, 40, 60) *n* values mixed dimensional and n = ∞ perovskites were grown on the glass substrates by depositing perovskite precursor solution, containing 1.35 M PbI_2_ adjusting MAI and the bulkier cation NMA amount stoichiometrically according to (NMA)_2_(MA)_n−1_PbnI_3n+1_ perovskite compound, where (NMA = 1-naphthylmethyl ammonium and MA = methyl ammonium), yielding the different dimensionality (*n*) values of perovskites. The precursor components were dissolved in DMF/DMSO (4:1) at followed by stirring with heating at 70 °C for 2 hours to obtain a clear solution. The glass substrates were cleaned with a diluted deacon soap solution followed by water and ethanol for 20 minutes each. Later the cleaned and dried substrates were treated with ozone plasma for 20 minutes before depositing perovskite film. One-step spin coating with anti-solvent dripping method was used to fabricate the perovskite films, with spinning rate of 5000 rpm for 13 second and diethyl ether as the antisolvent was dropped at 9 sec during spin coating step followed by annealing at 100 °C for 50 minutes to complete the crystallization of perovskite. The perovskite films so obtained have been used for further characterizations and thermal stability tests.

### Device fabrication

To fabricate mesoscopic architecture for *n* = ∞ and mixed dimensional perovskites, the patterned FTO (Pilkington TEC 15) glass substrates (1.5 cm × 2 cm) were obtained by etching with zinc powder and HCl (3 M). Later, substrates were cleaned with Hellmax soap solution, deionized water, and ethanol respectively by sonication. TiO_2_ compact layer precursor solution was prepared by mixing 600 µL of titanium diisopropoxide bis(acetylacetonate) (Sigma-Aldrich, 75% in 2-propanol) and 400 µL acetylacetone in 9 mL of isopropanol. The 40 nm thin compact layer was deposited by spray pyrolysis at 450 °C. The spray-coated TiO_2_ layer substrate was immersed in aqueous solution of 40 mM TiCl_4_ (Wako Pure Chemical Industries, >99%) for 30 minutes at 70 °C, rinsed with deionized water and dried at 500 °C for 30 minutes. Thin mesoporous layer (m-TiO_2_) of 150 nm was deposited over spray-coated compact film by spinning dilute solution of commercially available 30NR-T paste (Dysol) in ethanol (Sigma-Aldrich, ≥99.8%), with a ratio 5.5:1 w/w, then substrates were annealed at 500 °C for 30 minutes. Mesoporous films then were soaked into 40 mM of aqueous TiCl_4_ solution for 30 minutes at 70 °C followed by drying and sintered at 500 °C for 30 minutes. The photoactive layer coated over the m-TiO_2_ by spinning 1.35M precursor solution of perovskites using the same procedure as described for film formation. The hole transporting layer was deposited on the perovskite layer by using Spiro-OMATED in chlorobenzene (70 mg/mL) containing 20 µL tert-butyl pyridine, 15 µL of bis(trifluoromethane)sulfonimide lithium salt (520 mg/mL in acetonitrile) as well as 20 µL FK 209 Co(III) TFSI salt (50 mg/mL in acetonitrile) at 5000 rpm, 30 sec. Finally, the device fabrication procedure was completed by evaporating 100 nm gold as a top contact. The active area of cells fixed to 0.09 cm^2^.

### Stability test

To check the thermal stability of solar cells, the international standards for silicon solar cell reliability (climatic IEC 61646 chamber tests) necessitate testing at 85 °C^61^. Here, mentioned 85 °C represents the elevated temperature on the rooftop during hot summer days. The 85 °C temperature also falls in the range of reaction temperatures for the formation of most metal halide perovskites: 60–110 °C^62^. Thermal stability test of the perovskite films has been performed at 85 °C and 150 °C in dark and inert/vacuum atmosphere and of encapsulated devices at 85 °C for 360 hrs.

### Characterization

Current-Voltage (J-V) characteristics of the devices were measured by utilizing Keithley (model 2612A) digital source meter and an Oriel solar simulator (model 81172) equipped with 450 W xenon lamp which provides light spectral distribution of an AM 1.5G. Calibration for output power is performed by reference Si photodiode before the measurement. The active area for all the cells was maintained to 0.09 cm^2^. Incident photon-to-current efficiency (IPCE) measurement has been performed by utilizing a PVE300 (Bentham) equipped with dual light source Xenon/ quartz halogen lamp. The IPCE spectra were obtained in Dc mode under light with zero applied bias conditions. Absorption spectra and steady-state photoluminescence were recorded by using a UV–vis-NIR Spectrophotometer (SHIMADZU UV-3600) with an integrating sphere (ISR-3100) and Fluorolog-3 spectrofluorometric with 0.5 nm wavelength resolution respectively. Steady-state photoluminescence of the perovskite films on quartz was recorded by using a Fluorolog-3 spectrofluorometric with 0.5 nm wavelength resolution. Time-correlated single-photon counting (TCSPC) system was used for time-resolved photoluminescence (TRPL) with the laser pulsed power 405 nm, an excitation density of 4 µJ/cm^2^ with a repetition rate of 40 MHz (Acton SpectraPro 2300i, Princeton Instruments). Morphological and structural features of the films were obtained by using FESEM (JOEL JSM 6700 F) and X-ray diffractometer with Bragg–Brentano geometry (Bruker D8, Cu Kα source) armed with the divergent slit of 1° and Lynxeye strip detector respectively. The temperature-dependent *in-situ* glancing XRD measurements were carried out using the Bruker D8 Discover High resolution-XRD (Cu-Ka radiation operated at 40 kV and 40 mA) diffractometer, equipped with *in-situ* heating capability (Anton Paar DHS1100). ATR-FTIR measurement has been performed by using Frontier Perkin Erlmer instrument having optimized wavelength range 8300–350 cm^−1^, proprietary KBr beam splitter with spectral resolution 0.4 cm^−1^ for the 3028 cm^−1^ band methane. ^1^H and ^13^C NMR spectra of organic and hybrid perovskite compounds were recorded in DMSO-d_6_ solution using Bruker AV400 spectrometer.

## Supplementary information

Supplemetary informations.
