# Publisher Correction: Mixed-Dimensional Naphthylmethylammonium-Methylammonium Lead Iodide Perovskites with Improved Thermal Stability

**DOI:** 10.1038/s41598-020-65027-8

**Published:** 2020-05-20

**Authors:** Bhumika Chaudhary, Teck M. Koh, Benny Febriansyah, Annalisa Bruno, Nripan Mathews, Subodh G. Mhaisalkar, Cesare Soci

**Affiliations:** 1 Interdisciplinary Graduate School, Energy Research Institute @ Nanyang Technological University (ERI@N), Research Techno Plaza, X-Frontier Block, Level 5, 50 Nanyang Drive, 637553 Singapore, Singapore; 2 Energy Research Institute @ Nanyang Technological University (ERI@N), Research Techno Plaza, X-Frontier Block, Level 5, 50 Nanyang Drive, 637553 Singapore, Singapore; 3 School of Materials Science and Engineering, Nanyang Technological University, 50 Nanyang Avenue, 639798 Singapore, Singapore; 4 Division of Physics and Applied Physics School of Physical and Mathematical Sciences, Nanyang Technological University, 21 Nanyang Link, 637371 Singapore, Singapore

Correction to: *Scientific Reports* 10.1038/s41598-019-57015-4, published online 16 January 2020

The original version of this Article contained errors.

Firstly, there was a typographical error in the title and abstract.

“Mixed-Dimensional Naphthylmethylammoinium-Methylammonium Lead Iodide Perovskites with Improved Thermal Stability”

now reads:

“Mixed-Dimensional Naphthylmethylammonium-Methylammonium Lead Iodide Perovskites with Improved Thermal Stability”

“In this study, we show that by combining the organic ligand 1-naphthylmethylammoinium iodide (NMAI) with methylammonium (MA) to form a mixed dimensional (NMA)2(MA)n−1PbnI3n+1 perovskite the optical, crystallographic and morphological properties of the newly formed mixed dimensional perovskite films under thermal ageing can be retained.”

now reads:

“In this study, we show that by combining the organic ligand 1-naphthylmethylammonium iodide (NMAI) with methylammonium (MA) to form a mixed dimensional (NMA)2(MA)n−1PbnI3n+1 perovskite the optical, crystallographic and morphological properties of the newly formed mixed dimensional perovskite films under thermal ageing can be retained.”

In addition, an affiliation of Nripan Mathews was omitted. The correct affiliations for Nripan Mathews are listed below:

Energy Research Institute @ Nanyang Technological University (ERI@N), Research Techno Plaza, X-Frontier Block, Level 5, 50 Nanyang Drive, 637553, Singapore, Singapore

School of Materials Science and Engineering, Nanyang Technological University, 50 Nanyang Avenue, 639798, Singapore, Singapore

Furthermore, the Supplementary Information file accompanying this article contained a typographical error in Affiliation 2, which was incorrectly given as:

Energy Research Institute @ Nanyang Technological University (ERI@N), Research Techno Plaza, X-Frontier Block Level 550 Nanyang Drive, Singapore 637553.

The correct affiliation is listed below:

Energy Research Institute @ Nanyang Technological University (ERI@N), Research Techno Plaza, X-Frontier Block, Level 5, 50 Nanyang Drive, 637553, Singapore, Singapore.

These errors have now been corrected in the HTML and PDF versions of the Article, as well as in the Supplementary Information that accompanies this Article.

