# Supplementary material for: Mixed-Dimensional Naphthylmethylammoinium-Methylammonium Lead Iodide Perovskites with Improved Thermal Stability
Source: Sci Rep. 2020 Jan 16;10:429. doi: 10.1038/s41598-019-57015-4 (PMC6965185; doi:10.1038/s41598-019-57015-4)
Supplement: Supplementary file 1 — Supplemetary informations. [file 41598_2019_57015_MOESM1_ESM.docx]

Supplementary information (SI)

**Mixed-Dimensional Naphthylmethylammoinium-Methylammonium Lead Iodide Perovskites with Improved Thermal Stability**

*Bhumika Chaudhary^†^, Teck M. Koh ^‡,^ Benny Febriansyah^†^, Annalisa Bruno*^‡^, Nripan Mathews^§^, Subodh G. Mhaisalkar*^,‡ §^, Cesare Soci*^,#^*

^†^Interdisciplinary Graduate School, Energy Research Institute @ Nanyang Technological University (ERI@N), Research Techno Plaza, X-Frontier Block Level 550 Nanyang Drive, Singapore 637553

^‡^Energy Research Institute @ Nanyang Technological University (ERI@N), Research Techno Plaza, X-Frontier Block Level 550 Nanyang Drive, Singapore 637553

^§^School of Materials Science and Engineering, Nanyang Technological University
50 Nanyang Avenue, Singapore 639798

^#^Division of Physics and Applied Physics School of Physical and Mathematical Sciences, Nanyang Technological University, 21 Nanyang Link, Singapore 637371


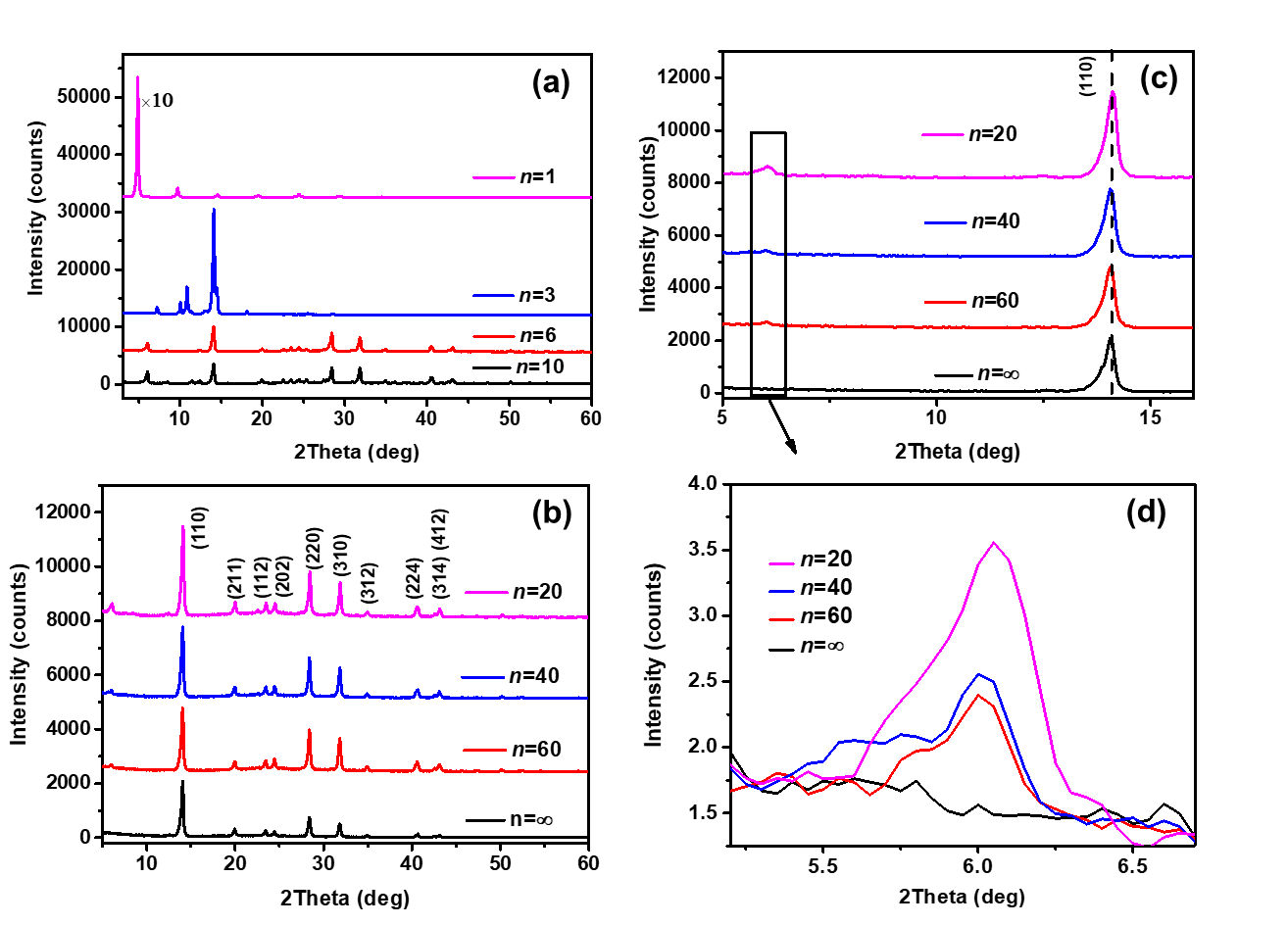


Figure S1: Glancing incident XRD patterns of (NMA)_2_(MA)*_n_*_-1_Pb*_n_*I_3_*_n_*_+1_ perovskite thin films. (a) *n*=1, *n*=3, 6, and 10; (b) *n*=20, 40, 60, ∞. Enlarged plots for the *n*=20, 40, 60, and ∞ perovskites: (c) highlighting the 13.4° peak corresponding to the 110-diffraction peak of the 3D structure and (d) the low angle (6.1°) diffraction peak related to the low dimensional phases.


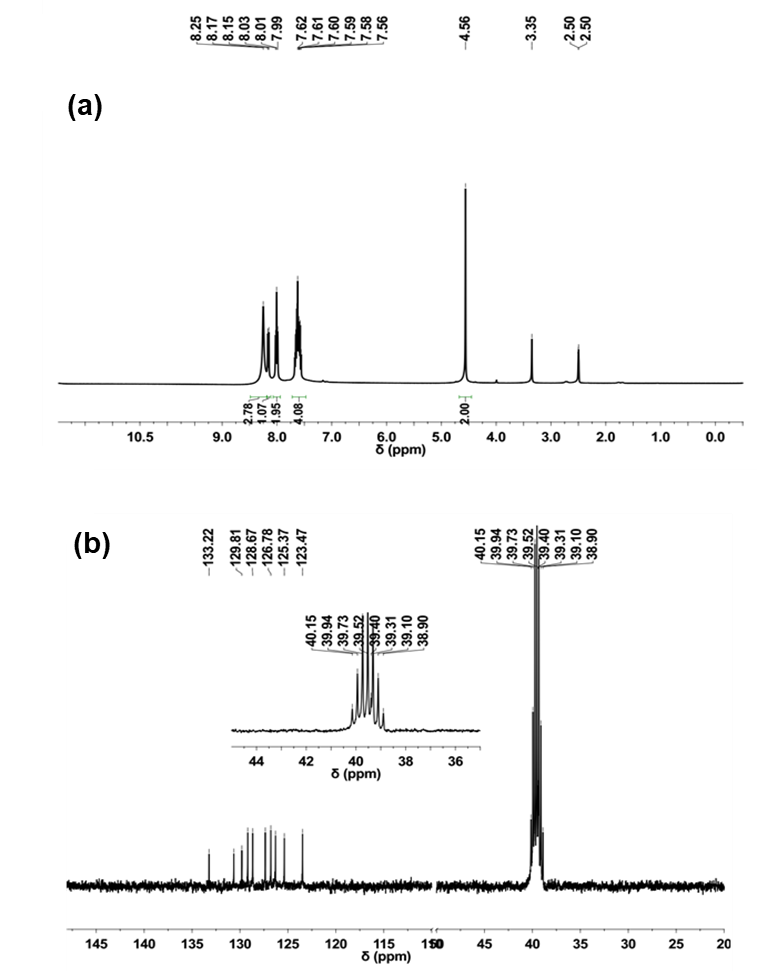


Figure S2: (a) ^1^H NMR spectra and (b) ^13^C NMR spectra at 298 K of naphthylmethylammonium iodide salt in deuterated dimethylsulfoxide (DMSO-d6). The assignment of the peaks is discussed in the Experimental Section of the manuscript.


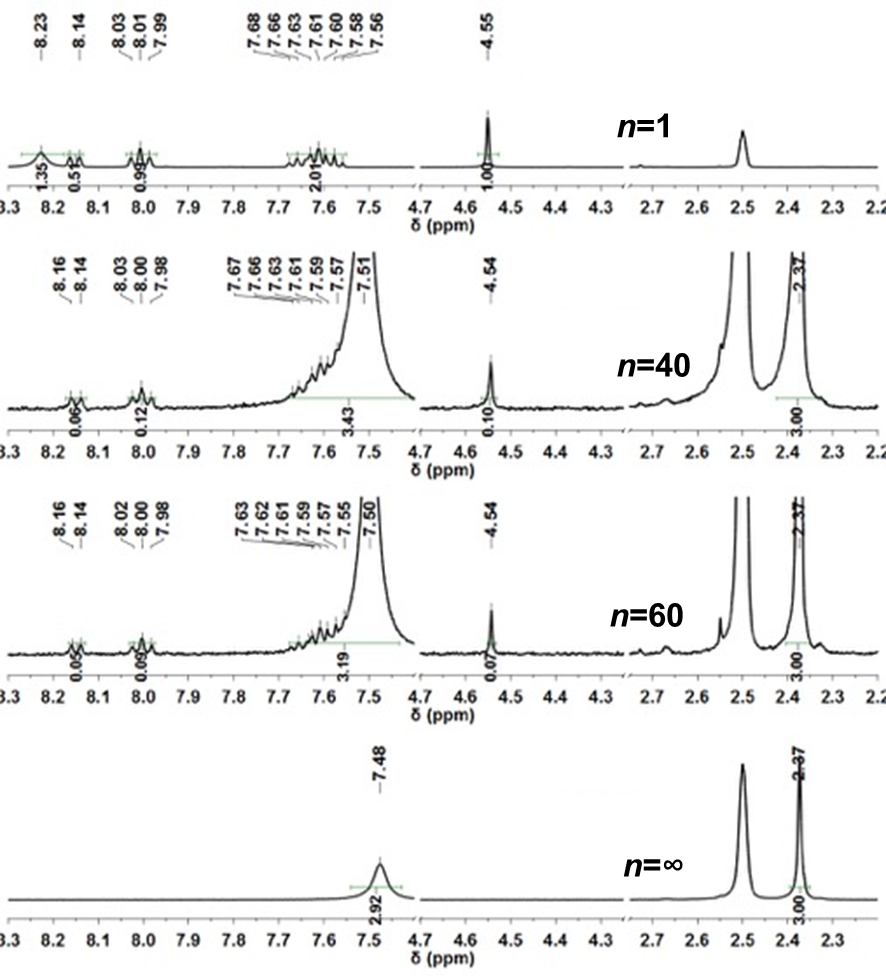


Figure S3: Room temperature (T=298 K) ^1^H NMR spectra of the perovskite samples (scraped from films) in deuterated dimethylsulfoxide (DMSO-d6) solvent containing the MAPbI_3_ and multidimensional perovskite (*n*=40, 60). The assignment of the peaks is discussed in the Results and Discussion Section of the manuscript. The mole ratio of NMA/MA for *n*=40 and *n*=60 is determined to be 1:20 and 1:29, respectively, based on the ratio of integrated peaks of methylene (CH_2_) protons in NMA and methyl (CH_3_) protons in MA, considering that one molecule of NMA and MA contains two and three protons.


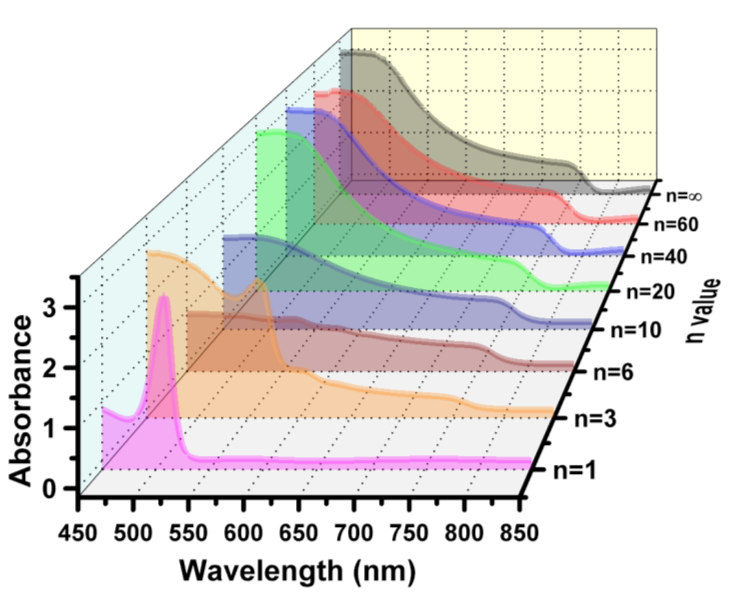


Figure S4: Absorption spectra of pristine perovskite films of different dimensionalities showing the optical band edge around 780 nm and the appearance of distinct excitonic absorption peaks in the 500-600 nm spectral region in low dimensional perovskites (*n*≤6).


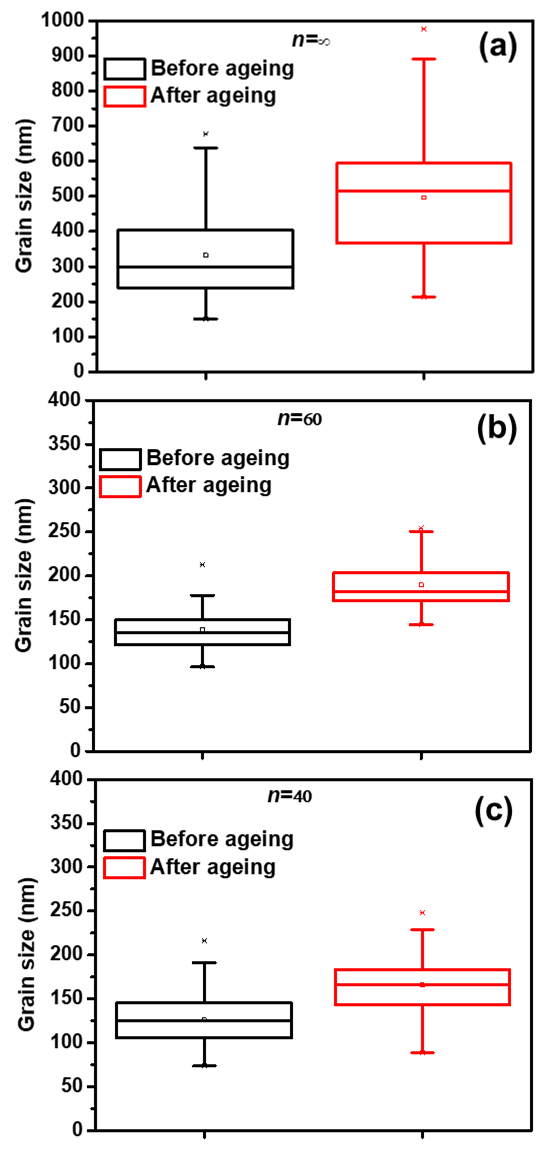


Figure S5: Grain size distributions in perovskite thin films before and after thermal ageing for 4hrs. for *n*=∞ (a) , *n*=60 (b) and *n*= 40 (c).

Table S1: Double exponential fitting parameters of time-resolved photoluminescence decay curves of pristine and aged (85°C for 4 hrs) perovskite films with different *n* values. The average fluorescence lifetime was calculated as τ_av_ = (τ_1*_a_1_+τ_2*_a_2_)/(a_1_+a_2_).

| ***n*** | **Film ageing condition** | **τ_1_ (ns)** | **a_1_ (%)** | **τ_2_ (ns)** | **a_2_ (%)** | **τ_av_ (ns)** | **Fluorescence lifetime reduction (%)** |
| --- | --- | --- | --- | --- | --- | --- | --- |
| ∞ | Pristine | 27.4 | 45.2 | 85.4 | 54.8 | 58.9 | 94.6 |
|  | Aged | 1.2 | 64.6 | 6.8 | 35.4 | 3.2 |  |
| 60 | Pristine | 31.4 | 38.5 | 101.1 | 61.5 | 74.6 | 77.0 |
|  | Aged | 6.1 | 64.4 | 31.3 | 35.6 | 17.0 |  |
| 40 | Pristine | 18.9 | 32.5 | 85.2 | 67.5 | 63.3 | 74.2 |
|  | Aged | 5.8 | 55.1 | 29.3 | 44.9 | 16.3 |  |


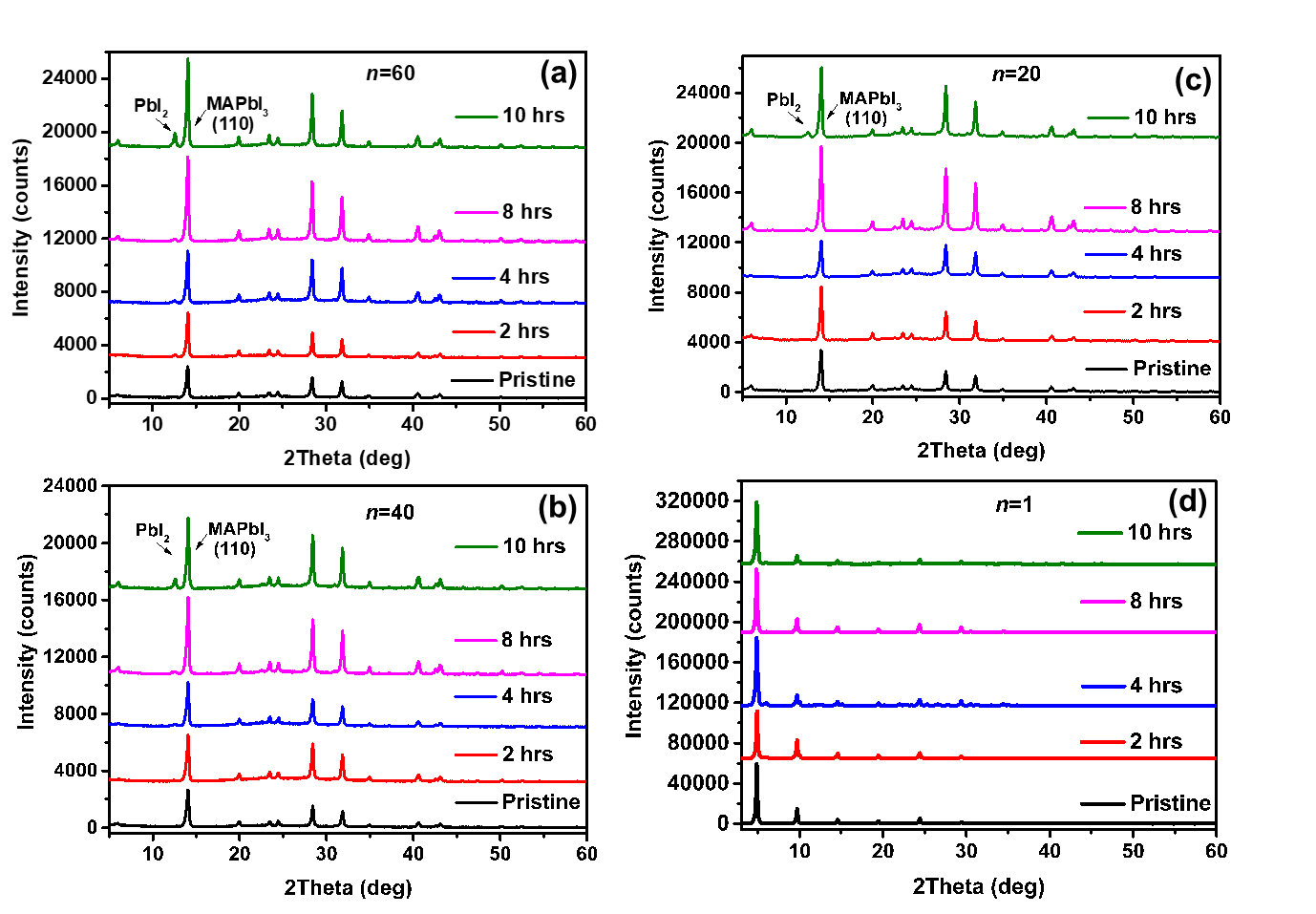


Figure S6: Time evolution of the glancing incident XRD spectra of perovskites thin films with different dimensionality during thermal ageing at 85˚C (in dark and inert atmosphere); a) *n*=60, b) *n*=40, c) *n*=20, and d) 2D perovskite films (pristine films were measured at room temperature).


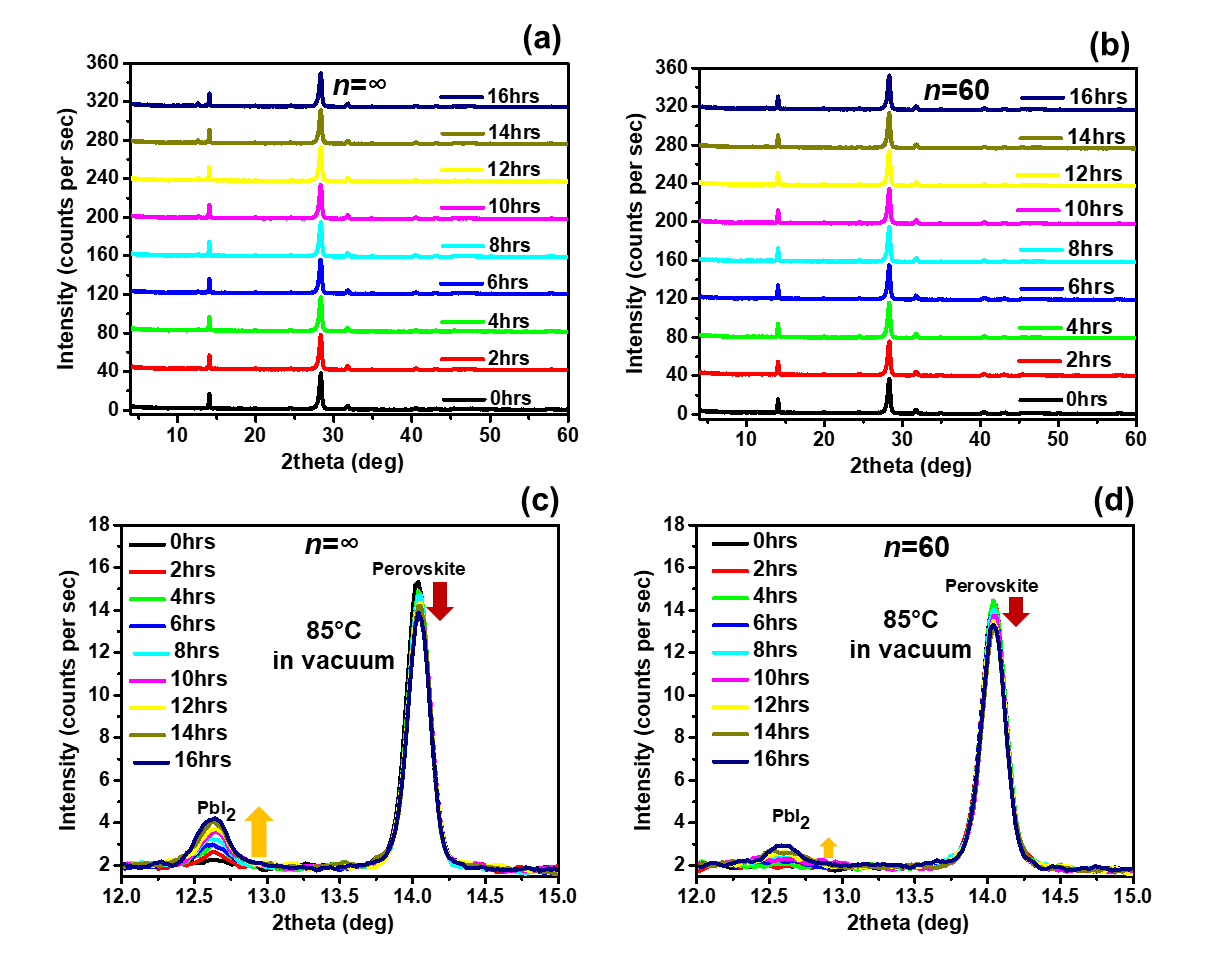


Figure S7: Glancing incident in-situ XRD patterns during 85 ^o^C isothermal ageing for *n*=∞ (a) and *n*=60 (b) perovskites. XRD patterned zoomed between 12 ^o^ -25^o^ for *n***=∞** (c) and n=60 (d).


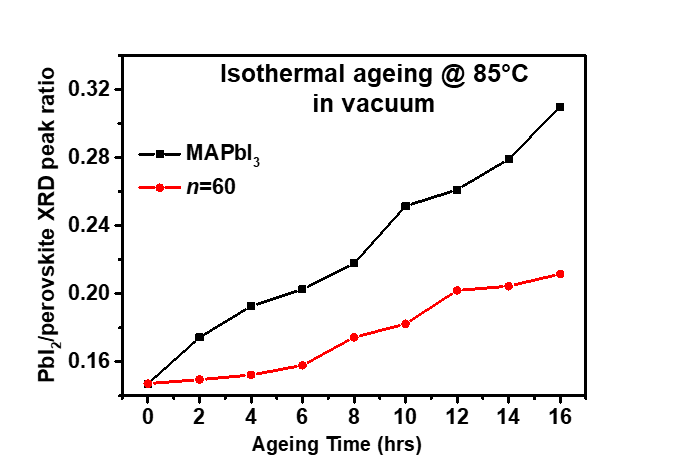


Figure S8: Ratio between the PbI_2_ diffraction peak (2θ=12.7˚) and the main perovskite diffraction peak (2θ=14.2˚) intensities during 85^o^C isothermal ageing over 16 hours for *n*=∞ and *n*= 60 perovskite thin films extracted from XRD pattern of figure S7 (c) *n*=∞ and S7 (d) *n*=60.


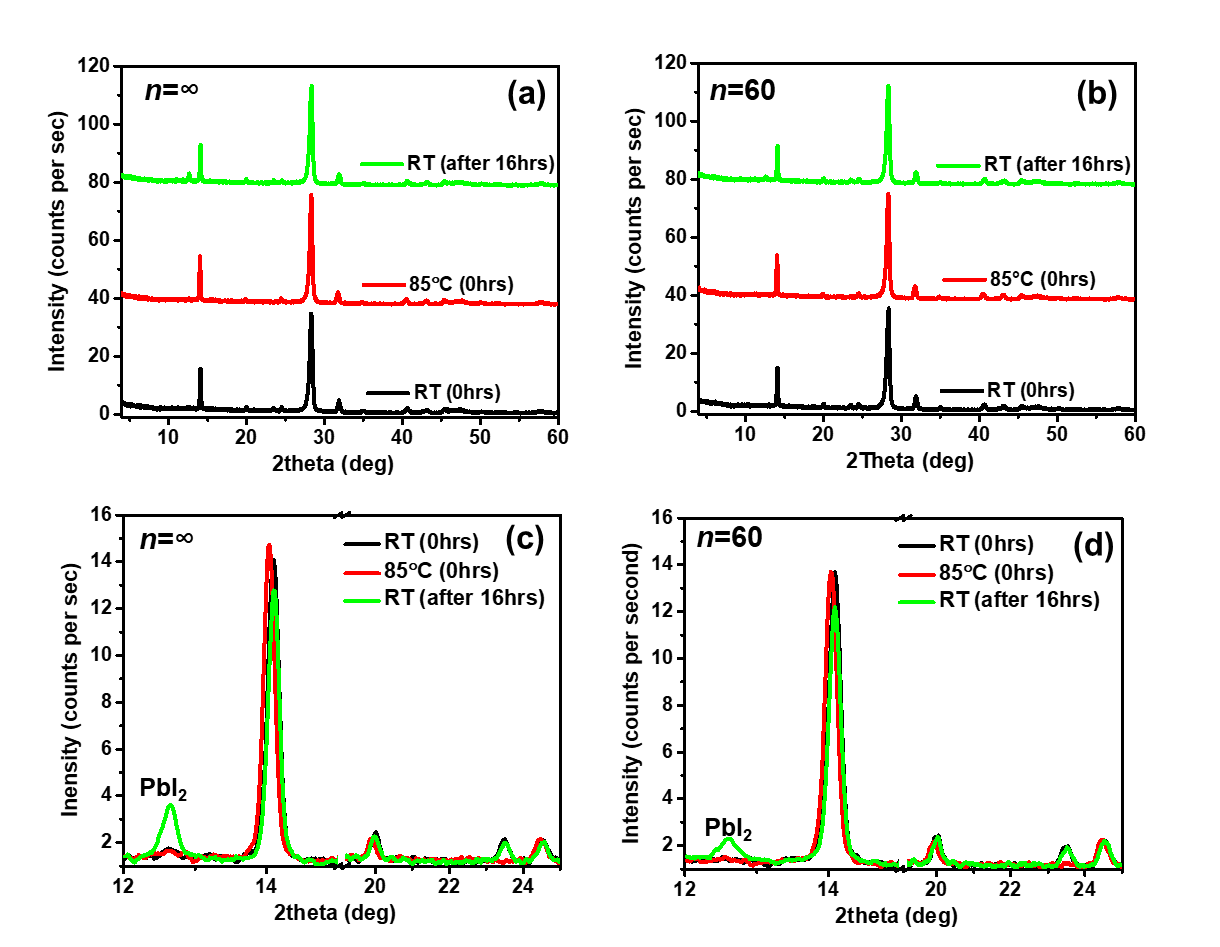
Figure S9: XDR patterns at room temperature (RT), at 85 ^o^C and at RT after 16 hours isothermal ageing at 85^o^C for *n***=∞** (a) and n=60 (b). XRD patterns zoomed in the 12 ^o^ -25^o^ for *n***=∞** (c) and *n*=60 (d).


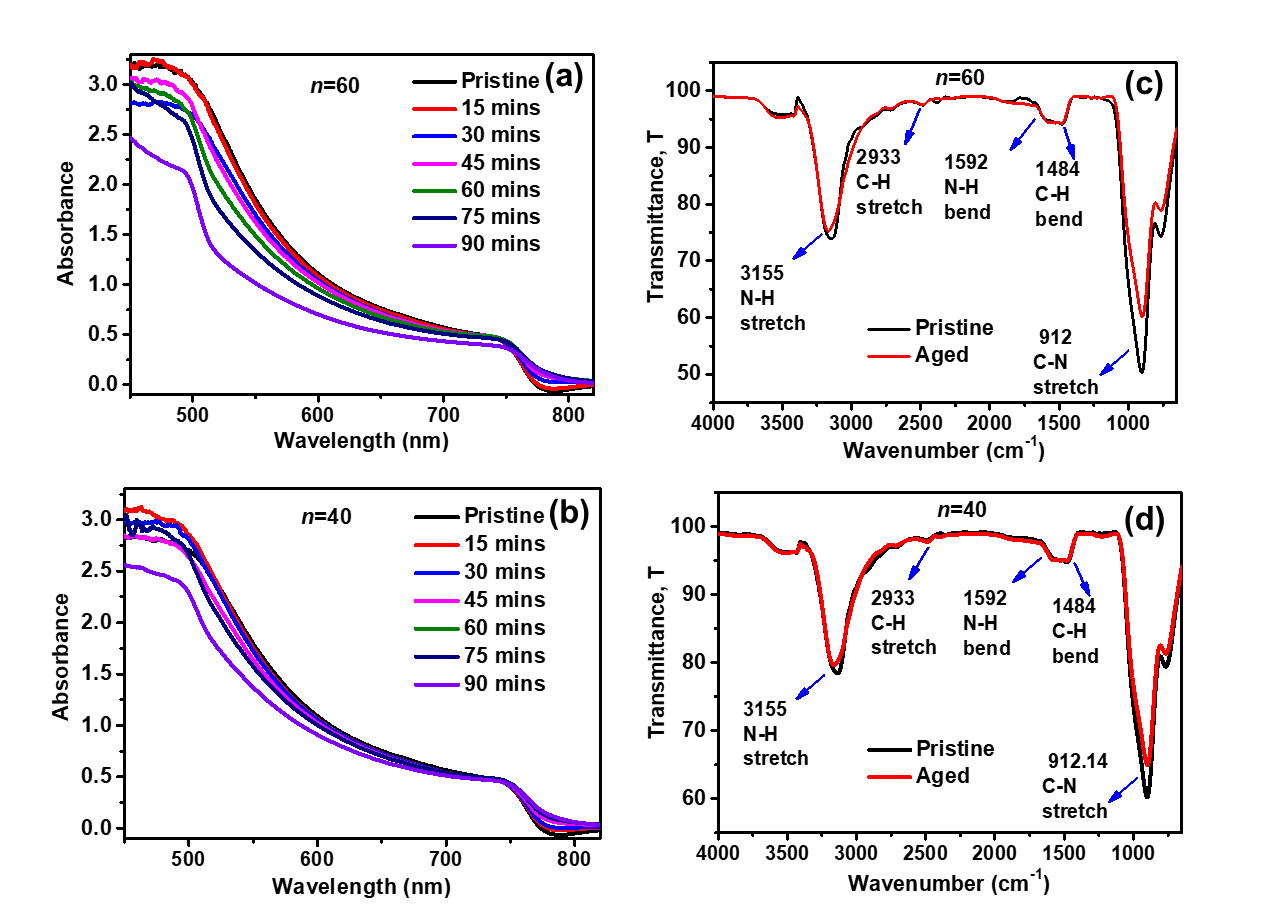


Figure S10: Optical and vibrational signatures of thermal ageing of high order *n* perovskites (*n*=60, 40). a-b) Time evolution of the absorption spectra of films heated at 150˚C and c-d) variation of the vibrational modes of pristine and aged (85°C for 4 hrs) films, measured by FTIR-ATR spectroscopy.


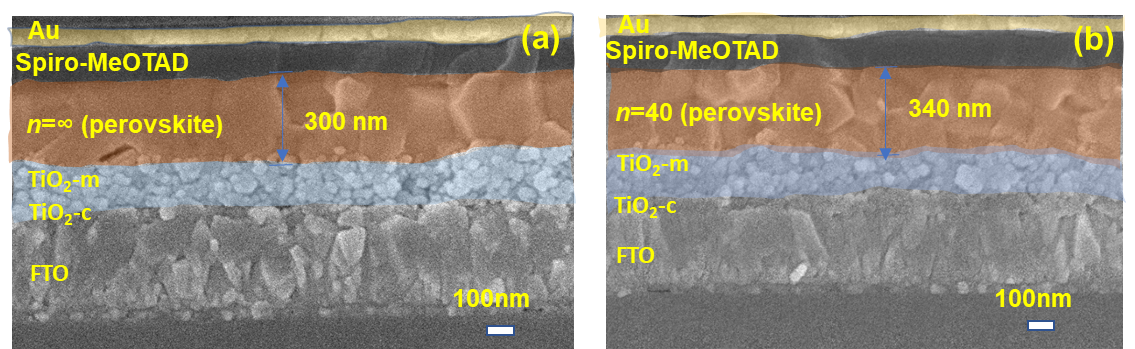


Figure S11: Cross-sectional SEM images of solar cell structures with high-dimensional perovskite active layers. a) *n*=∞ MAPbI_3_ and b) *n*=40 NMA-MA perovskite.

**
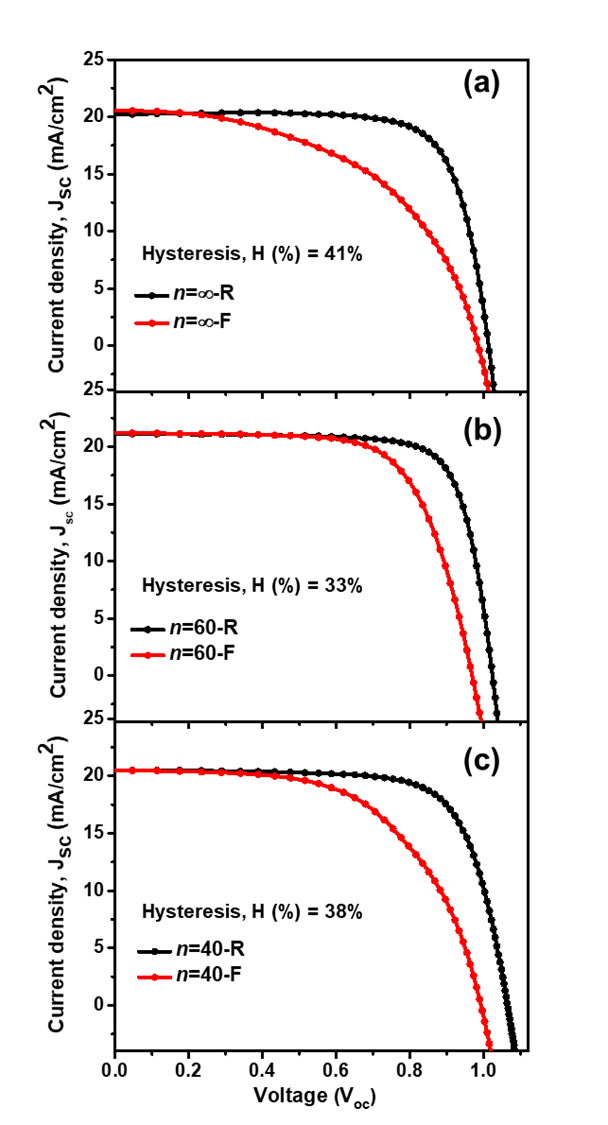
**

Figure S12: Forward and reverse scans J-V characteristics of best-performing perovskite solar cells. a) n=∞, b) n=60 and c) n=40. Hysteresis percentage was calculated as, H (%) = 100 * (1 – (J_intergral reverse_ /J_intergral forward_))%.^61^

**
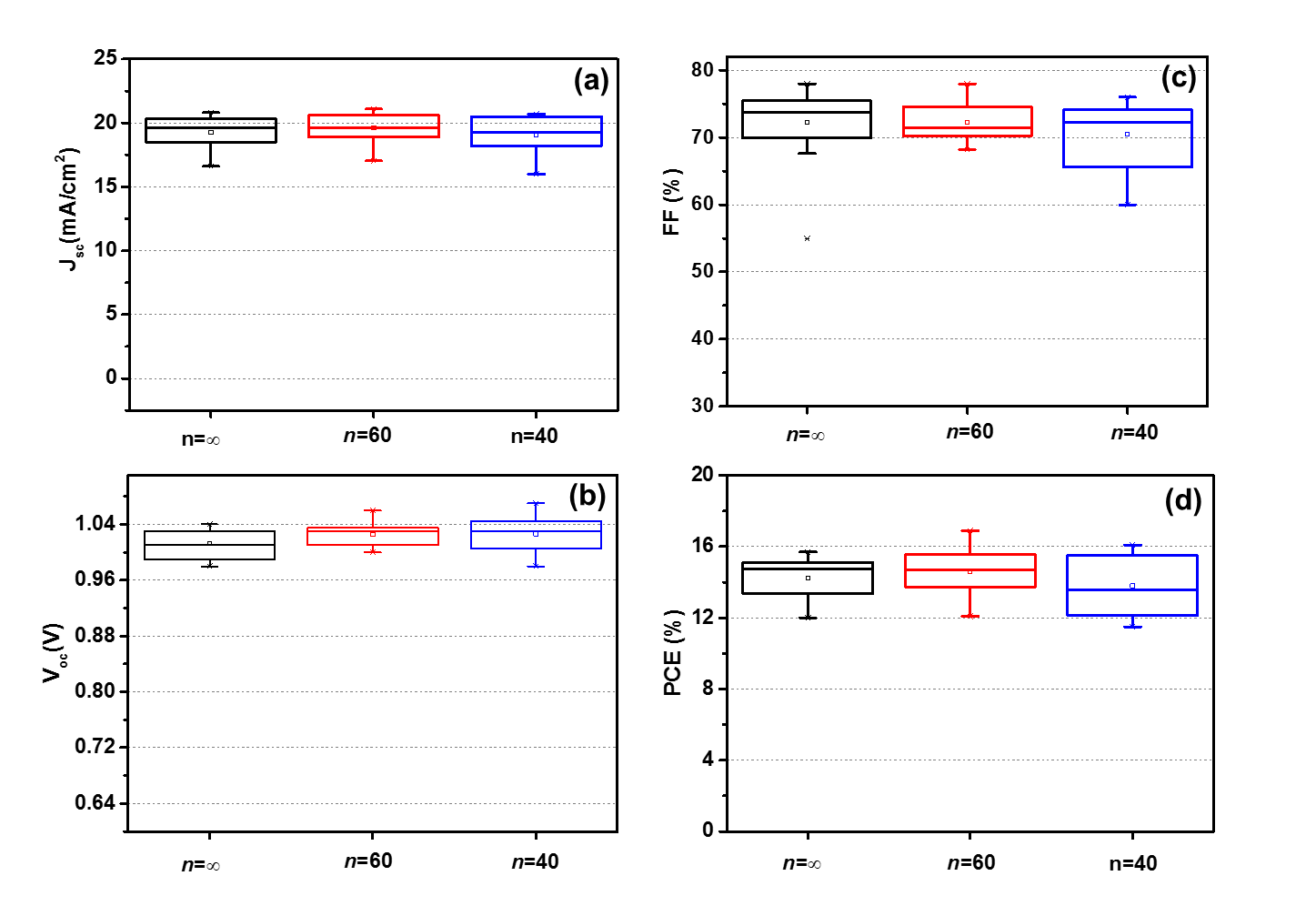
**

Figure S13: Solar cell characteristics. a) Short circuit current density (J_sc_), b) open-circuit voltage (V_oc_), c) fill factor (FF) and d) power conversion efficiency (PCE) distribution of total 20 perovskite solar cells with perovskite active layers of different dimensionality (*n*=∞, 60, 40).
